# A CT-based technique to predict optimal projection for self-expanding TAVI in patients with different aortic valve anatomies

**DOI:** 10.1186/s12872-021-02387-7

**Published:** 2021-12-07

**Authors:** Xi Wang, Fei Chen, Tian-Yuan Xiong, Yi-Jian Li, Yuan-Weixiang Ou, Qiao Li, Yong Peng, Jia-Fu Wei, Sen He, Mao Chen, Yuan Feng

**Affiliations:** Department of Cardiology, West China Hospital, Sichuan University, No.37 Guo Xue Xiang, Chengdu, Sichuan 610041 People’s Republic of China

**Keywords:** TAVI, MSCT, Optimal projection, Bicuspid aortic valve

## Abstract

**Background:**

Optimal projection is essential for valve deployment during transcatheter aortic valve implantation (TAVI). The purpose of this study was to propose an approach to predict optimal projection in TAVI candidates with different aortic valve anatomies.

**Methods:**

331 patients undergoing self-expanding TAVI were included and the so-called non-coronary cusp (NCC)-parallel technique was utilized, which generated the predicted projection by connecting NCC commissures on the transverse plane on the pre-procedural computed tomography images.

**Results:**

37.8% of the study cohort were bicuspid aortic valve (BAV) patients. Around 80% of both NCC-parallel views and final views were in the right anterior oblique (RAO) and caudal (CAU) quadrant. There was less than 5° change required from the NCC-parallel view to the final implanted view in 79% of tricuspid aortic valve (TAV) patients but only in 27% (13/48) of type 0 BAV patients with coronary arteries originated from the different cusps. After excluding the above mentioned BAV patients, 62.3% (48/77) of BAV patients needed less than 5° change to achieve optimal projection and only in 8 patients, the angular change was larger than 10° in either left/right anterior oblique or cranial/caudal direction.

**Conclusions:**

The NCC-parallel technique provides reliable prediction for optimal projection in self-expanding TAVI in all TAV and most BAV patients, with a vast majority of views in the RAO and CAU quadrant.

**Supplementary Information:**

The online version contains supplementary material available at 10.1186/s12872-021-02387-7.

## Background

Optimal projection views are critical to transcatheter aortic valve implantation (TAVI) procedures during transcatheter heart valve (THV) deployment, in order to eliminate the parallax of both the annulus and delivery catheter, which is of vital importance to achieve adequate perception of implantation depth and reduced risk of complications such as paravalvular leak (PVL) as well as conduction disturbances [1].

In the expert consensus from the Society of Cardiovascular Computed Tomography (SCCT) focusing on CT imaging in TAVI, it is strongly recommended that prediction of optimal projection based on pre-procedural multislice computed tomography (MSCT) be provided for each individual patient [2], which helps operators find the optimal views quickly during the procedure without performing multiple aortographies [3–5]. The 3-cusp co-planar view with the right coronary cusp (RCC) projected between the non-coronary cusp (NCC) and left coronary cusp (LCC) is a typical and commonly used CT-based projection view [6]. However, it is highly possible that the catheter of the self-expanding device is not aligned in the 3-cusp view during TAVI procedure, resulting in a period of time to search for another LAO view to remove the parallax to the catheter.

Recently a cusp-overlap technique was suggested for self-expanding TAVI projections and has been gaining popularity in TAVI community [7–9]. On the cusp-overlap view, the RCC and LCC hinge points would be overlapped, while the NCC would be isolated at the opposite annulus border. However, the determination of cusp hinge points is quite subjective according to the imager’s choice and especially difficult when there’s severe calcification at the cusp bottom. In addition, since TAVI is expanding to treat more young patients, in whom bicuspid aortic valve (BAV) is a common anatomy [10], Heart Teams would face more BAV patients in the future. The optimal projection for BAV patients is warranted to achieve satisfying clinical results in this cohort.

Herein, we propose a pre-procedural CT-based approach to predict optimal projection views during self-expanding TAVI and aim to utilize this method in patients with different aortic valve anatomies.

## Methods

### Study population

This study was conducted prospectively among patients undergoing transfemoral self-expanding TAVI between January 2019 and October 2020 at West China Hospital, Sichuan, China. After excluding patients with previous aortic valve replacement (n = 9) and poor CT imaging quality (n = 2), a total of 331 patients were included in the final cohort. Decisions to proceed TAVI were confirmed after thorough discussion by the Heart Team. Three domestic self-expanding devices were implanted including Venus A-Valve (Venus Medtech, Hangzhou, China), VitaFlow Valve (Shanghai MicroPort CardioFlow Medtech, Shanghai, China) and TaurusOne Valve (Peijia Medical, Suzhou, China). Procedural complications and outcomes were defined according to the Valve Academic Research Consortium-2 criteria classification [11]. The study was approved by the Institutional Ethical Committee of West China Hospital, Sichuan University and was conducted in accordance with the principles of the Declaration of Helsinki. All patients gave written informed consent.

### CT acquisition

Pre-procedural MSCT were performed in all patients using a 256-slice system (Revolution CT, GE, Boston, Massachusetts or SOMATOM Definition Flash CT, Siemens Medical Solutions, Forchheim, Germany). The contrast-enhanced CT scan was acquired with collimation of 256*0.625 mm. Tube current was modified automatically according to the patient’s size at 100–120 kV. Iopamidol (Sine, Shanghai, China) was injected intravenously with 50–100 ml at a flow rate of 4–6 ml/s. Image acquisition was performed with electrocardiographic gating. All the CT data were reconstructed using images in the systolic phase (25%-35% intervals throughout the cardiac cycle) with a slice thickness and a slice increment both of 0.625 mm and analyzed using FluoroCT 3.0 (Circle Cardiovascular Imaging Inc., Calgary, Canada) by two independent physicians (Y. Feng and F. Chen).

### Optimal projection for TAVI

**Fig. 1:**
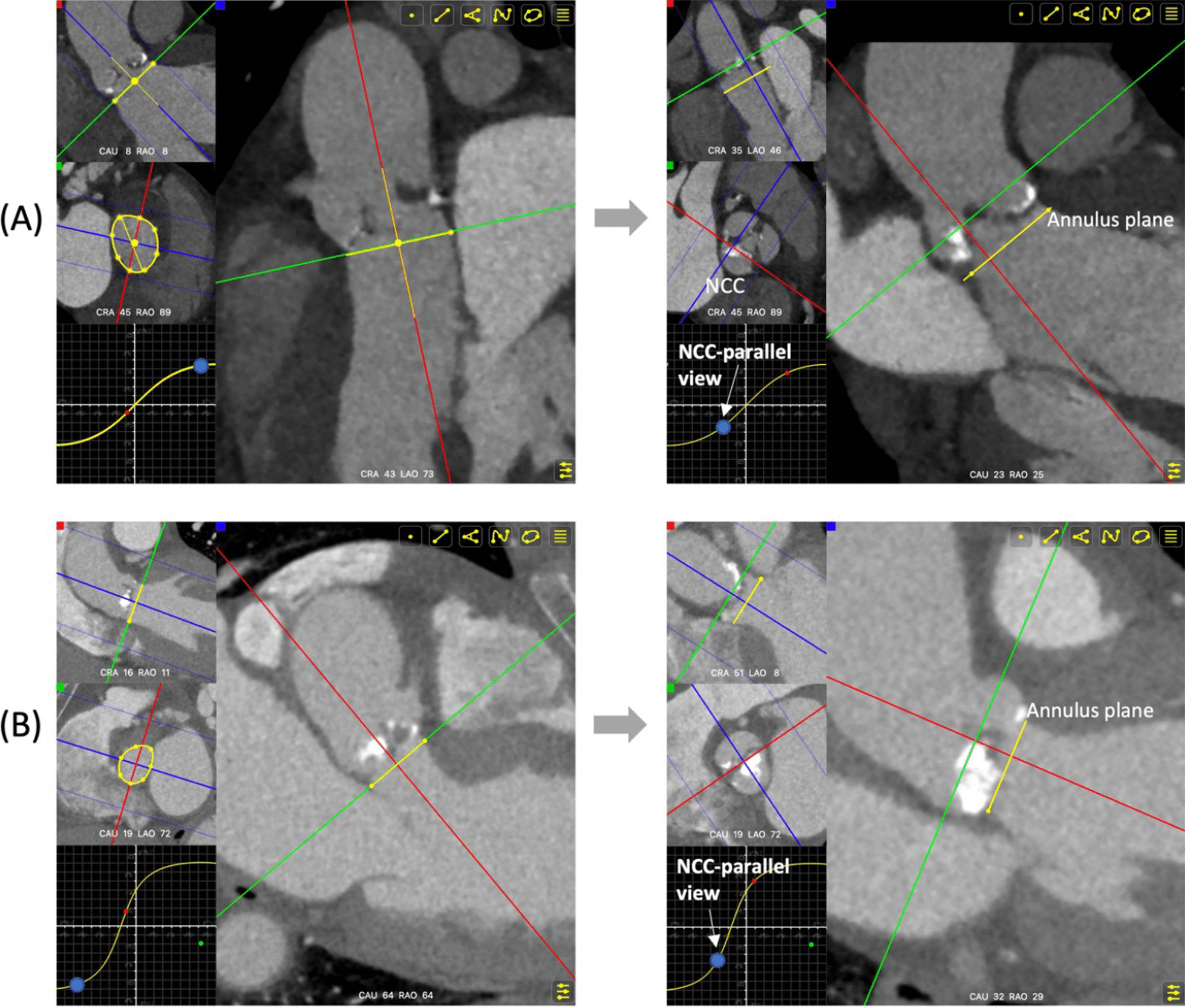
The non-coronary cusp-parallel technique to predict optimal projection during transcatheter aortic valve implantation. **a** After identifying the native annulus, the S-curve of the annulus would be generated by FluoroCT software, on which the annulus would always be aligned. Then one plane parallel to the annulus where all commissures could be clearly observed would be chosen to determine the non-coronary cusp (NCC) -parallel view. The S-curve is tracked until the red line (sagittal plane) connects the R-N commissure and L-N commissure on the determined transverse plane, showing the NCC-parallel view as right anterior oblique (RAO) 25 and caudal (CAU) 23. **b** As for type 0 bicuspid aortic valve patients, commissures between the two cusps would be connected by the red line when tracking the annulus S-curve, showing the NCC-parallel view as RAO 29 and CAU 32

As the delivery catheter is pushed through the descending aorta to the ascending aorta, the catheter would progress following the course of the aorta. Finally, the delivery system would be naturally positioned toward the outer curvature of the aortic root and land in the commissure between the right and non-coronary cusp (R-N commissure). As a consequence, the tangent line to the NCC commissures (R-N commissure and L-N commissure) is parallel to the projection of delivery catheter on the annulus plane, and almost parallel to the THV annulus plane in common anatomic structures. Therefore, we propose a novel approach to predict optimal projection in self-expanding TAVI and name it as NCC-parallel technique. After identifying the native annulus, one plane parallel to the annulus where all commissures could be clearly observed would be chosen to determine the NCC-parallel view. The S-curve of the annulus generated by FluoroCT software, on which the annulus would always be aligned, is tracked until the red line (sagittal plane) connects the R-N commissure and L-N commissure on the determined transverse plane (Fig. 1a, Additional file 1). By applying this technique, the RCC would not be centered, resulting in a totally different view from the conventional 3-cusp view.

The aortic valve anatomy would also be identified on pre-procedural CT imaging. The type of BAV was determined according to Sievers’ classification [12]. The NCC-parallel technique could be applied in patients with tricuspid aortic valve (TAV) and type 1 BAV, irrespective of the raphe location. As for type 0 BAV patients, commissures between the two cusps would be connected by the red line (sagittal plane) on the transverse plane (Fig. 1b).

The operator would adjust the C-arm to the predicted projection view during TAVI after the crimped THV crossed the aortic valve. As this technique may not provide precise projection prediction for patients with uncommon anatomy including horizontal aorta and some types of BAVs, there could still be residual parallax of the catheter on fluoroscopy. Then further C-arm adjustment was needed to achieve no parallax (three radiopaque markers on the THV aligned) before valve deployment. The final fluoroscopy view under which the valve deployment started was defined as the optimal projection view. Then a routine and careful TAVI would be executed.

To investigate the difference between the NCC-parallel view and cusp-overlap view, pre-procedural CT images from the most recent 100 patients in this cohort (type 0 BAVs were excluded) were retrospectively assessed to determine the cusp-overlap view following the instructions described before [9].

### Statistical analysis

Continuous variables with normal distribution were described as mean ± SD and compared using Student t test. Those not showing normal distribution were reported as median (25th, 75th percentile) and compared by Wilcoxon rank sum test. Categorical data were presented as frequencies with percentages and analyzed using chi-square test, fisher exact test or cochran-armitage trend test. Scatter plots were illustrated with left anterior oblique (LAO)/ right anterior oblique (RAO) angles as the x-axis and cranial (CRA)/ caudal (CAU) angles as the y-axis to show the overlapping of NCC-parallel views and final implanted views, and furthermore, the angular changes from NCC-parallel views to final implanted views. SPSS version 26 (IBM Corporation, Armonk, New York) was used to perform the analyses. Analyses were considered significant at a 2-tailed p value < 0.05.

## Results

### Baseline characteristics

As for the baseline features for the study cohort including 331 patients undergoing self-expanding TAVI, the mean age was 74 years old with 60% of the patients as male. The mean Society of Thoracic Surgeons score was 5.09%. Patients with BAV were younger (72.09 ± 7.17 vs. 74.54 ± 7.33 years, p = 0.003) and identified with higher aortic valve calcium volume (730.81 ± 580.64 vs. 362.16 ± 371.76 mm^3^, p < 0.001) when compared to patients with TAV. Baseline comorbidities were demonstrated in Table 1.

**Table 1:** Characteristics of the study population

	All (n = 331)	TAV (n = 206)	BAV (n = 125)	P value
Age, years	73.61 ± 7.35	74.54 ± 7.33	72.09 ± 7.17	0.003
Male, % (n)	59.8 (198)	62.6 (129)	55.2 (69)	0.18
Body Mass Index, kg/m^2^	22.88 ± 3.60	22.71 ± 3.70	23.16 ± 3.43	0.28
NYHA III-IV, % (n)	62.5 (207)	60.2 (124)	66.4 (83)	0.26
LVEF < 50%, % (n)	37.5 (124)	36.9 (76)	38.4 (48)	0.78
STS score, %	5.09 ± 5.12	5.36 ± 5.73	4.65 ± 3.91	0.23
Aortic valve calcium volume, mm^3^	525.30 ± 508.45	362.16 ± 371.76	730.81 ± 580.64	< 0.001
Comorbidities, % (n)				
Hypertension	49.5 (164)	52.4 (108)	44.8 (56)	0.18
Diabetes	19.9 (66)	18.4 (38)	22.4 (28)	0.38
Coronary artery disease	22.4 (74)	22.8 (47)	21.6 (27)	0.80
Prior myocardial infarction	1.8 (6)	2.4 (5)	0.8 (1)	0.52
Prior stroke	4.5 (15)	3.9 (8)	5.6 (7)	0.47
COPD	11.8 (39)	14.6 (30)	7.2 (9)	0.04
Chronic kidney disease	6.9 (23)	6.8 (14)	7.2 (9)	0.89
Atrial fibrillation	18.7 (62)	21.8 (45)	13.6 (17)	0.06
Prior pacemaker	3.0 (10)	3.9 (8)	1.6 (2)	0.40

*NYHA* New York Heart Association, *LVEF* left ventricular ejection fraction, *STS* The Society of Thoracic Surgeons, *COPD* chronic obstructive pulmonary disease

### Predicted and final implanted views

**Fig. 2:**
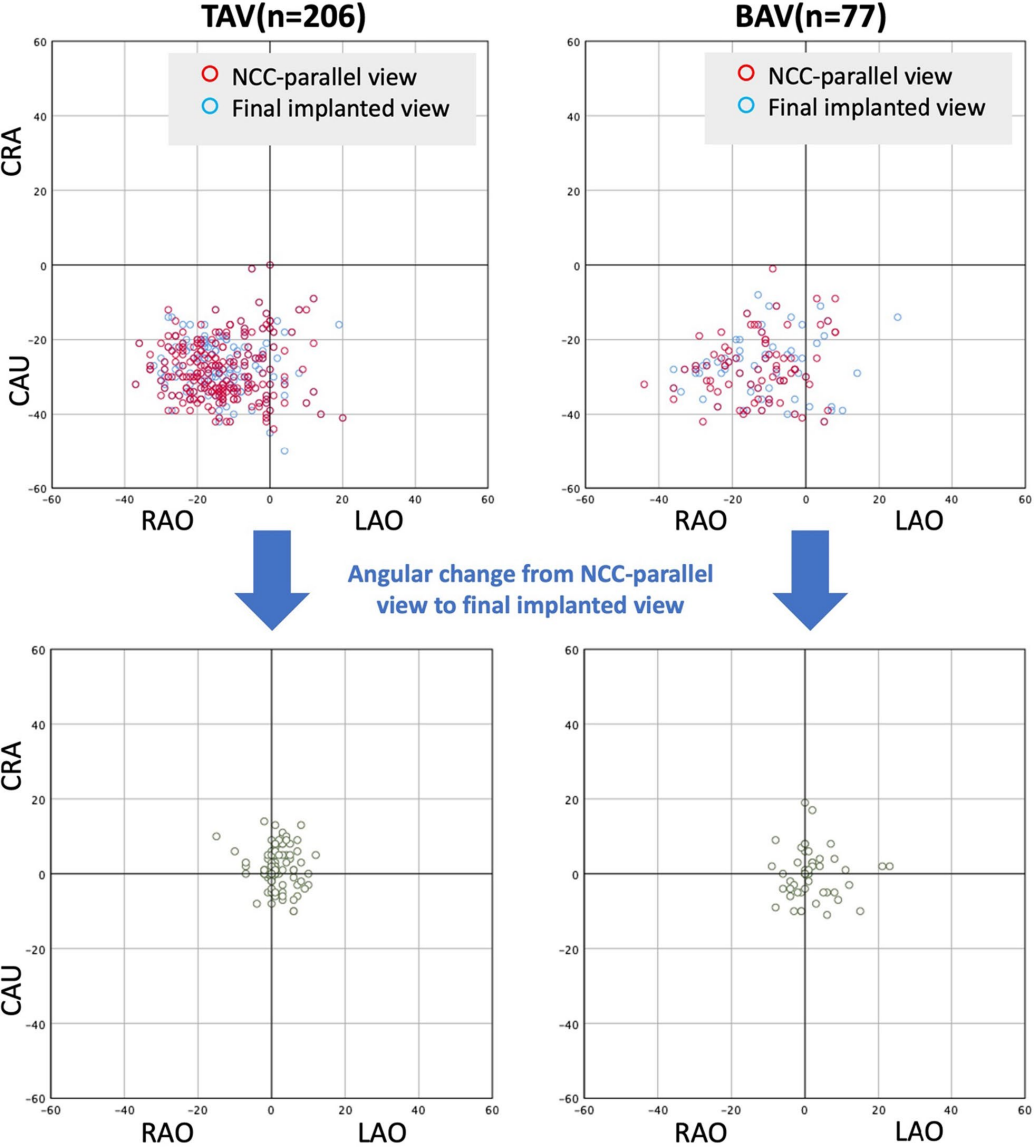
Comparison of NCC-parallel view and final implanted view. The NCC-parallel view and final implanted view could most often be obtained in the RAO/CAU quadrant for both TAVs and BAVs (type 0 subtype 2 BAVs were excluded). There was less than 5° change required from NCC-parallel view to the final implanted view in 79% of TAV patients and 62.3% of BAV patients. BAV, bicuspid aortic valve; CAU, caudal; CRA, cranial; LAO, left anterior oblique; NCC, non-coronary cusp; RAO, right anterior oblique; TAV, tricuspid aortic valve

Among all patients, 37.8% (n = 125) showed bicuspid anatomy on pre-procedural CT, which consisted of 56 type 1 BAVs, 21 type 0 BAVs with coronaries originated from the same cusp (subtype 1) and 48 type 0 BAVs with coronaries originated from different cusps (subtype 2). Around 80% of both NCC-parallel views and final implanted views were in the RAO/CAU quadrant. As for accuracy for prediction of this technique, there was less than 5° change required from NCC-parallel view to the final implanted view in 79% of TAV patients (n = 163) but only in 27% of type 0 subtype 2 BAV patients (n = 13). After excluding patients with type 0 subtype 2 BAV, 62.3% (48/77) of BAV patients needed less than 5° change to achieve optimal projection and only in 8 patients, the angular change was larger than 10° in either LAO/RAO or CRA/CAU direction (Fig. 2).

**Fig. 3:**
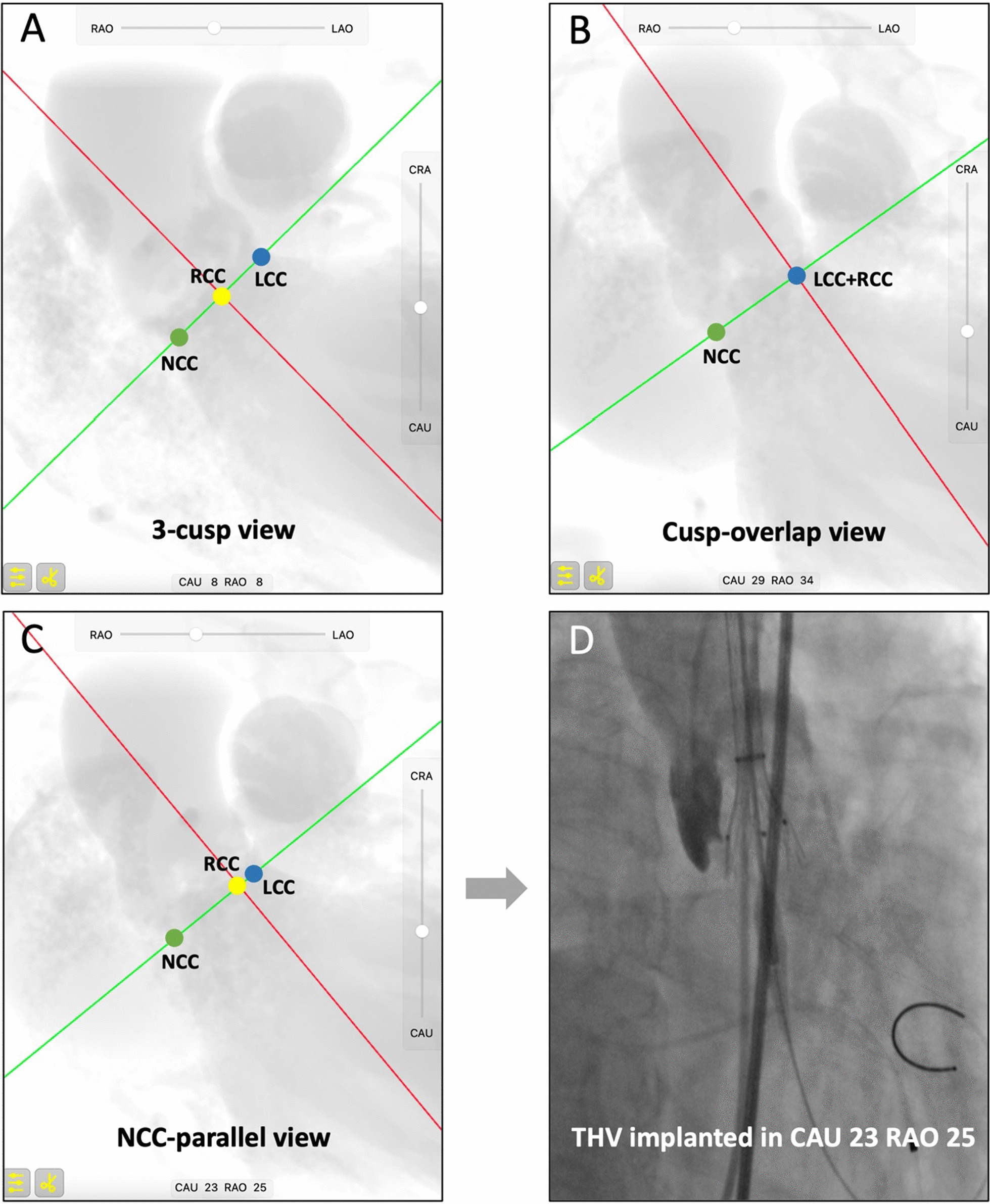
Different predicted views and the final implanted view. The conventional 3-cusp co-planar view (RAO8 CAU8) (**a**), cusp-overlap view (RAO34 CAU29) (**b**) and NCC-parallel view (RAO25 CAU23) (**c**) in one patient. THV was deployed in the NCC-parallel view (**d**) and there was no parallax of the delivery catheter as the three radiopaque markers on the catheter were in plane. CAU, caudal; LCC, left coronary cusp; NCC, non-coronary cusp; RAO, right anterior oblique; RCC, right coronary cusp; THV, transcatheter aortic valve

The NCC-parallel view was compared with the cusp-overlap view in 100 patients (77 TAVs and 23 type 1 BAVs). The proportion of patients with a cusp-overlap view or an NCC-parallel view in the RAO/CAU quadrant was 96% and 94%, respectively. The two views differed less than 10° in 69% of patients and the cusp overlap view was calculated to be more RAO/CAU as compared to the NCC-parallel view in 71% of patients. Here also demonstrated an example to show the 3-cusp co-planar view, cusp-overlap view and NCC-parallel view in one patient and the fluoroscopy image during initial valve deployment (Fig. 3).

### Procedural outcomes

The THV was successfully implanted in all patients. Incidence of major procedural complications including major vascular complications, major bleeding and stroke were all lower than 2%. Only 2 patients were observed with more than moderate PVL after TAVI. The new permanent pacemaker implantation (PPMI) rate was 24.8% in this study population (Table 2).

**Table 2:** Procedural outcomes of the study population

Procedural outcomes, %(n)	All(n = 331)	TAV(n = 206)	BAV(n = 125)
Successful THV implantation	100 (331)	100 (206)	100 (125)
Major vascular complication	2 (7)	2.4 (5)	1.6 (2)
Major bleeding	1.2 (4)	0.5 (1)	2.4 (3)
New onset permanent pacemaker	24.8 (82)	27.2 (56)	20.8 (26)
In-hospital stroke	0.3 (1)	0.5 (1)	0
Moderate or severe paravalvular leak	0.6 (2)	0.5 (1)	0.8 (1)

*THV* transcatheter heart valve

## Discussion

In this study, by assuring that the crimped valve would land in the R-N commissure, we proposed a novel pre-procedural CT-based technique to predict the optimal projection during self-expanding TAVI. This NCC-parallel technique was utilized in over 300 patients and demonstrated high accuracy in TAV patients compared to final implanted projection, on which the parallax of delivery catheter would surely be removed. As for BAV patients, close proximity to the final projection was also observed in most patients, while the feasibility was limited in patients with type 0 BAV and two coronaries originated from different cusps. Thus, this technique could help TAVI operators find optimal projections easily and rapidly in all TAV and most BAV patients, which only requires C-arm fine tune during the procedure, potentially reducing fluoroscopy time as well as contrast volume [4].

In the SCCT expert consensus, typical views which were mentioned included the conventional 3-cusp co-planar view, the predefined LAO angulations or the view to visualize the left main stem [2]. These views are usually achieved with LAO angles [13]. On LAO projections of the left heart, both the coronary ostia and native valve orifice could be clearly observed, however, the left ventricular outflow tract (LVOT) as well as delivery catheter would be foreshortened, which leads to misjudgment of implantation depth. Ben-Shoshan et al. retrospectively analyzed the pre-procedural CT images using the FluoroCT software in 100 patients to determine the cusp-overlap views [9]. They’ve shown that over 80% of the cusp-overlap projections were in the RAO and CAU quadrant. Besides eliminating the parallax of catheter, RAO and CAU views provide maximal elongation of LVOT as well as catheter, and also better visualization of the NCC, of which the hinge point is the anatomic deepest position of the sinus, therefore, clear estimation of implantation depth could be achieved [7]. Previous studies have confirmed that implantation depth > membranous septum (MS) length is an independent predictor for PPMI after TAVI [14, 15]. Since the HIS-bundle bifurcates at the lower edge of MS and then becomes left bundle branch beneath the NCC [14, 16], RAO and CAU views would allow a high valve implantation relative to the conduction system and potentially reduce the risk of post-TAVI conduction disturbances.

In our study, around 80% of both NCC-parallel views and final implanted projections were RAO and CAU views in 331 patients undergoing self-expanding TAVI, which is consistent with previous findings for the cusp-overlap technique [7, 9]. It is worth noting that we firstly investigated the prediction of optimal projection in patients with various BAV morphologies, and the results of a group of BAV patients using RAO and CAU views were firstly reported. The NCC-parallel technique provided close prediction of optimal projection for type 0 BAV patients with coronary arteries originated from the same cusp, which is not applicable in the cusp-overlap technique. However, our technique cannot be utilized in patients with type 0 BAV and two coronaries originated from different cusps. We suppose the reason is that there might be anatomic transposition of the two cusps and orifice orientation change to satisfy coronary artery blood flow, and then the position of the catheter becomes unpredictable [17, 18]. Further studies should be focused on proposing the approach to predict optimal projection for this specific aortic valve anatomy.

Even though the annulus plane is decided by cusp hinge points, as one plane contains countless points, the mild difference of hinge point location may not result in the change of annulus plane but could lead to significantly different cusp-overlap view, as the cusp-overlap technique highly relies on the precise location of the cusp hinge points. In our NCC-parallel technique, simply connecting R-N commissure and L-N commissure on the plane parallel to the annulus could generate the predicted projection views. This technique has been confirmed to be highly reliable in TAV patients. Larger angular difference between the final and predicted views was shown in BAV patients compared to TAV patients. This could be explained by the fact that severer calcification on the leaflets of BAV might affect the identification of the commissures. Therefore, the NCC-parallel technique would possibly be suggested when the cusp hinge points are hardly precisely distinguished because of annular calcification while the cusp-overlap technique seems more suitable for patients with severe leaflet calcification involving the commissures. Besides, the cusp overlap view was reported to be more RAO/CAU as compared to the NCC-parallel view in our study. Therefore, when the cusp-overlap angulation is too extreme for the C-arm to reach, changing to the NCC-parallel view might be an alternative.

The current study also firstly showed the clinical outcomes of patients undergoing TAVI using this novel technique. The device was successfully implanted in all patients with a very low rate of severe complications, including major vascular complications, major bleeding, stroke and more than moderate PVL. As for new onset PPMI after TAVI, we noted a similar incidence compared with that in recent multicenter studies using newer-generation self-expanding devices [19–21]. Sammour et al. recently found that deploying the balloon-expandable SAPIEN 3 valve in RAO and CAU views could better achieve higher implantation than in conventional co-planar views, which results in significant reduction in post-TAVI PPMI [1]. Further studies are needed to investigate the reduction of PPMI rate after applying the NCC-parallel technique, in order to confirm the clinical importance of RAO and CAU projections.

### Limitations

Although we prospectively applied this novel technique to patients undergoing TAVI, the present study only demonstrated single-center experience with Chinese domestic devices. Further investigations are necessary to test the NCC-parallel technique in other TAVI systems in various heart centers. Besides, when defining the final fluoroscopy view for valve deployment as the optimal projection, the catheter parallax was fully eliminated, which was considered as crucial to visualize the implantation depth to the NCC. However, this maneuver possibly resulted in slight misalignment of the native annulus. In addition, there would be inevitably slight discrepancy of the CT-based predicted view on fluoroscopy owing to different patient position during CT acquisition and the TAVI procedure. Last but not least, we only showed clinical characteristics with a patient cohort using this technique. Large retrospective studies as well as prospective randomized studies should be conducted to identify the clinical benefits of the NCC-parallel technique by comparison with conventional 3-cusp views, especially in reducing implantation depth and PPMI rates.

## Conclusion

The NCC-parallel technique provides easy, rapid and reliable prediction based on pre-procedural CT for optimal projection views in self-expanding TAVI in all TAV and most BAV patients, with a vast majority of views in the RAO and CAU quadrant.

## Supplementary Information

**Additional file 1**. A step-by-step video about how to obtain NCC-parallel view. Identify the annulus—show the annulus S-curve—define a plane parallel to annulus to observe the commissures—let the red line cross NCC commissures when tracking the S-curve—double check the commissure location—finished.

## Data Availability

The datasets used and/or analysed during the current study are available from the corresponding author on reasonable request.

## Abbreviations

BAV: Bicuspid aortic valve
CAU: Caudal
COPD: Chronic obstructive pulmonary disease
CRA: Cranial
LAO: Left anterior oblique
LCC: Left coronary cusp
LVOT: Left ventricular outflow tract
MS: Membranous septum
MSCT: Multislice computed tomography
NCC: Non-coronary cusp
PPMI: Permanent pacemaker implantation
PVL: Paravalvular leak
RAO: Right anterior oblique
RCC: Right coronary cusp
SCCT: Society of Cardiovascular Computed Tomography
STS: The Society of Thoracic Surgeons
TAV: Tricuspid aortic valve
TAVI: Transcatheter aortic valve implantation
THV: Transcatheter heart valve
